# Compressive Learning for the Classification and Reconstruction of Synthetic Aperture Radar Data

**DOI:** 10.3390/s25216508

**Published:** 2025-10-22

**Authors:** Mobina Keymasi, Omid Ghozatlou, Miguel Heredia Conde, Mihai Datcu

**Affiliations:** 1 Research Center for Spatial Information (CEOSpaceTech), The National University of Science and Technology POLITEHNICA Bucharest, 060042 Bucharest, Romania; omid.ghozatlou@upb.ro (O.G.); mihai.datcu@upb.ro (M.D.); 2 Center for Sensor Systems (ZESS), University of Siegen, Paul-Bonatz-Straße 9–11, 57076 Siegen, Germany; heredia@zess.uni-siegen.de

**Keywords:** compressive learning (CL), synthetic aperture radar (SAR), signal processing, machine learning (ML)

## Abstract

Compressive learning (CL) for synthetic aperture radar (SAR) aims to reduce the volume of data required for effective SAR image processing while preserving classification performance and minimizing reconstruction loss. This study introduces a novel CL framework comprising three distinct scenarios: (I) direct classification from compressed measurements, (II) image reconstruction from compressed measurements, and (III) joint classification and reconstruction using a trainable compression layer. The proposed network includes a linear transformation layer that performs data compression, followed by multilayer perceptrons (MLPs) tailored for classification and reconstruction tasks. In the joint scenario, end-to-end training enables the compression layer to learn task-specific representations that improve both inference and data recovery. We evaluate our approach on the MNIST and MSTAR datasets across various compression ratios. Experimental results show that joint training significantly improves classification accuracy and reconstruction quality compared to fixed compression schemes. These findings highlight the potential of adaptive compressive learning for enhancing SAR data processing efficiency.

## 1. Introduction

Remote sensing (RS) has become an integral component of modern Earth observation (EO), enabling advancements in critical domains such as environmental monitoring, resource management, agricultural planning, and urban development [1]. RS systems are broadly categorized into optical and microwave sensing technologies, which are the foundation of satellite-based EO platforms. These systems are further divided into two types: passive sensors, which rely on external energy sources like sunlight, and active sensors, which generate their own signals for data acquisition [2]. Among active sensors, synthetic aperture radar (SAR) stands out for its ability to capture high-resolution images independent of weather conditions or time of day. By emitting microwave signals, SAR not only penetrates cloud cover but also operates effectively in darkness, making it an indispensable tool for continuous monitoring in challenging environments. These attributes allow SAR to overcome the limitations of optical imaging, particularly in scenarios where adverse weather or lighting conditions hinder data collection.

The integration of machine learning (ML) techniques into RS systems has significantly enhanced their analytical capabilities, transforming how EO data are processed and interpreted. ML enables the automated extraction of meaningful insights from large, complex datasets acquired by diverse sensors, facilitating more efficient and accurate analyses [3,4]. These techniques have been pivotal in tasks such as land cover classification, anomaly detection, and environmental parameter estimation, all of which are crucial for informed decision-making in EO applications [5,6]. However, SAR data present unique challenges for ML-driven analysis due to their reliance on microwave backscatter, which results in distinctive texture patterns and pervasive speckle noise. These characteristics demand specialized algorithms capable of effectively capturing and processing the intricate details of SAR imagery, distinguishing it from traditional optical data processing approaches [7,8].

Despite SAR’s significant advantages, processing its unique data poses several challenges. The data’s complex backscattering patterns, high dimensionality, and pervasive speckle noise demand innovative solutions for efficient compression and analysis. Traditional CS-based methods often fall short due to their reliance on fixed sensing matrices, which are not optimized for the diverse textures and scattering phenomena inherent in SAR imagery [6,9,10]. Furthermore, most conventional approaches treat sampling, reconstruction, and classification as independent tasks, leading to suboptimal results when applied to SAR’s dynamic scenarios.

Compressive sensing (CS) provides a transformative solution to the data-intensive challenges of SAR imaging. In contrast to traditional sampling methods that require a complete dataset for accurate representation, CS allows for the acquisition of significantly fewer measurements while preserving the essential features of the original data [11,12,13]. This capability reduces the volume of data that need to be transmitted, stored, and processed, offering substantial benefits in RS applications, particularly in scenarios with limited bandwidth and storage resources. These advantages are especially critical in EO, where maintaining high data fidelity and ensuring efficient processing are paramount. The integration of CS with ML, known as compressive learning (CL), has further expanded its potential by enabling inference directly on compressed data. This approach eliminates the need for full data reconstruction, resulting in faster, more efficient, and cost-effective RS operations [14,15,16].

The application of compressive learning to SAR data faces several unique challenges. SAR imagery contains complex backscattering patterns and distinctive scattering behaviors that make traditional compression methods less effective [17]. The inherent speckle noise in SAR images requires specialized handling during compression to preserve signal fidelity [18,19]. Additionally, SAR systems generate massive volumes of high-dimensional data, making efficient compression crucial for practical applications [6].

These challenges necessitate the development of advanced compressive learning frameworks that can effectively integrate sampling, reconstruction, and classification while minimizing information loss.

The integration of CL with SAR data introduces several specialized research challenges and opportunities. Implementing CL in the context of SAR requires the development of unique measurement strategies, sparse representations, and advanced reconstruction techniques tailored to the distinctive characteristics of SAR imagery [20]. SAR images exhibit high sensitivity to environmental conditions and sensor parameters, leading to unique noise patterns, backscatter phenomena, and complex scattering behaviors. These attributes pose significant challenges for conventional CS techniques, which are typically optimized for optical or standard image types [18,19,21]. To address these complexities, researchers have proposed advanced methods such as the shearlet transform, which more effectively captures the intrinsic geometry of SAR data compared to traditional approaches [21,22]. Additionally, techniques like the directional lifting wavelet transform (DLWT) and Bayesian-based models have been developed to enhance SAR image reconstruction by reducing speckle noise and preserving fine structural details within compressed data representations [19,23].

Despite significant advancements in CS applications for SAR, the use of CL principles at the sensing stage remains underexplored. Integrating CL directly into SAR systems offers the potential to enhance data processing by reducing the volume of raw data collected and focusing on capturing only the most informative measurements. This approach holds promise for developing next-generation SAR instruments optimized for onboard data compression and processing. In response to this gap, the present study proposes a CL framework specifically tailored for SAR, employing joint training to combine sampling and reconstruction tasks within a single, cohesive network. The proposed method utilizes a trainable sensing matrix that simultaneously learns to optimize classification and reconstruction accuracy. Through joint training, the model effectively captures SAR-specific features and minimizes information loss at the sensing stage.

The proposed CL framework is evaluated using a comparative analysis of three experimental configurations designed to explore the trade-offs between data compression, classification, and reconstruction. These scenarios include (I) direct classification using a fixed sensing matrix, (II) signal reconstruction from compressed measurements, and (III) a joint classification–reconstruction architecture with a trainable sensing matrix. The third scenario introduces a fully learnable compression layer that jointly optimizes classification accuracy and reconstruction fidelity during training. This approach not only addresses the limitations of fixed sensing matrices but also adapts to the distinctive structural features of SAR data. Through extensive experimentation, we demonstrate the performance benefits of this unified, task-aware framework in both general (MNIST) and SAR-specific (MSTAR) contexts.

This paper is organized as follows: Section 2 presents the detailed methodology, elaborating on the design of the sampling matrix, the network architecture, and the integration of classification and reconstruction. Section 3 describes the experimental setup, including datasets, training procedures, and performance metrics. Section 4 and Section 5 discuss the results, analyze the contributions of the proposed method, and draw conclusions, respectively, with insights into future directions for compressive SAR learning.

## 2. Literature Review

Synthetic aperture radar (SAR) is widely recognized for its ability to operate in diverse environmental conditions, offering all-weather and day-and-night imaging capabilities. While SAR’s unique backscattering properties enable high-resolution imaging, they also introduce challenges such as speckle noise and complex scattering phenomena. Addressing these challenges has been the focus of several studies, particularly through advanced processing techniques. For instance, Xu et al. [6] proposed a sparse SAR imaging framework using compressed sensing (CS) and machine learning (ML) principles to address data sparsity and noise challenges. Similarly, sparse representation-based SAR imaging methods, such as the work by Cetin et al. [24], leverage sparsity-driven algorithms to achieve data-efficient SAR imaging. While effective in reducing data requirements, these methods lack integration with classification tasks [25].

In the context of noise reduction, Hou et al. [19] utilized Bayesian compressive sensing in the directional lifting wavelet transform domain to mitigate speckle noise while preserving fine image details. Although effective for denoising, the method did not integrate classification capabilities, which are critical for many Earth observation (EO) tasks. Similarly, Giusti et al. [26] compared CS-based methods with state-of-the-art super-resolution techniques for inverse SAR (ISAR) image reconstruction, showing moderate improvements in resolution but falling short in computational efficiency. Recent advancements have also explored sparsity-driven and machine learning-based super-resolution techniques, such as those proposed by Zhang et al. [6] , which focus on improving image resolution and denoising through adaptive learning. However, these methods often prioritize reconstruction fidelity over joint optimization with classification tasks.

The integration of ML with CS, referred to as compressive learning (CL), has introduced new paradigms for SAR data processing by enabling inference directly on compressed data without full reconstruction. Patel et al. [15] demonstrated the utility of compressed SAR for target recognition, showcasing its potential to reduce data storage and transmission requirements. However, their framework lacked a trainable sensing mechanism, which limits adaptability across different SAR applications. Similarly, Machidon et al. [27] explored deep learning-based SAR target classification on compressed data, demonstrating improved classification accuracy but without incorporating trainable sensing matrices.

Recent studies have explored trainable sensing matrices to optimize both sampling and reconstruction. For instance, Lee et al. [28] developed a deep learning-based CS framework for automotive SAR systems, achieving higher reconstruction fidelity but not addressing classification tasks. Wei et al. [16] further advanced parametric super-resolution SAR reconstruction using deep neural networks. While their model demonstrated superior resolution capabilities, it did not explore joint optimization of sampling and classification tasks, a gap that our framework addresses. Additionally, they proposed an adaptive CS framework incorporating deep neural networks for SAR imaging, which improved reconstruction accuracy under diverse scenarios but did not address classification integration [29].

SAR data often demand advanced processing techniques for effective analysis. Shearlet transforms, as proposed by Easley et al. [21], have been utilized to capture SAR’s geometric features more effectively than traditional approaches. Similarly, Bo et al. [30] explored the despeckling of SAR images using shearlet transforms, demonstrating improved visual clarity. Despite these advancements, these methods focus exclusively on reconstruction and lack integration with classification tasks, a critical requirement in many operational EO scenarios. Other studies, such as Amin [31], explored the combination of CS and sparsity-aware transforms for SAR image enhancement but focused primarily on reconstruction without considering downstream tasks like classification.

While significant advancements have been made in CS, ML, and SAR, several limitations persist in existing approaches, hindering their ability to fully exploit SAR’s unique data characteristics. One critical shortcoming is the fragmented optimization of sampling, reconstruction, and classification tasks. Most existing frameworks treat these processes as independent steps, which often results in inefficiencies and suboptimal performance, especially in dynamic and real-world SAR scenarios [6,19]. The lack of integration among these tasks limits their ability to leverage synergies, thereby failing to achieve holistic improvements in SAR data processing.

Another major limitation lies in the restricted adaptability of existing methods. Many current approaches rely on predefined transformations or fixed sensing matrices, which, while effective in controlled conditions, lack the flexibility needed to handle diverse and evolving SAR datasets. This rigidity reduces the applicability of such models in complex EO tasks that demand context-aware and data-driven adaptability [15,26].

Furthermore, few studies have explored joint training frameworks that optimize sampling, reconstruction, and classification simultaneously. This gap represents a missed opportunity to improve overall performance by tailoring the data acquisition process to downstream tasks. For instance, while some works have demonstrated the potential of deep learning for SAR reconstruction, they often overlook the benefits of a unified framework that integrates these processes into a cohesive optimization pipeline [16,28].

The proposed CL framework addresses these gaps by introducing a novel joint training approach. By leveraging a trainable sensing matrix, the framework seamlessly integrates sampling, reconstruction, and classification tasks into a single end-to-end model. This design not only enhances classification accuracy but also minimizes information loss during data acquisition, ensuring robust and efficient performance across diverse SAR scenarios. Compared to existing methods, the proposed framework demonstrates superior adaptability and efficiency, setting a new benchmark for SAR data processing in EO applications.

## 3. Methodology

In this section, we present the proposed compressive learning (CL) methodology, which combines data sampling, compression, and reconstruction in a unified framework tailored for synthetic aperture radar (SAR) imagery. SAR images, distinct from optical data, contain complex backscattering patterns and high noise levels, especially due to the speckle effect inherent in microwave imaging. Addressing these challenges requires a methodology that preserves geometric features and textural details while achieving efficient data compression.

**Figure 1:**
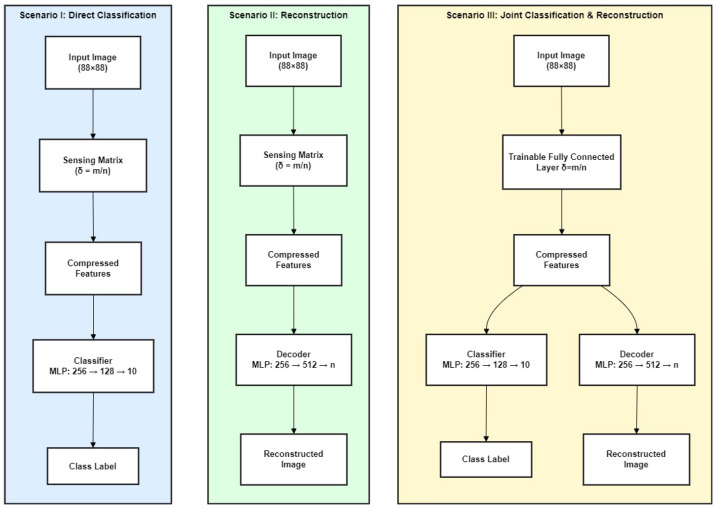
Overview of the three proposed compressive learning scenarios. (I) Direct Classification: Compressed features obtained from a fixed sensing matrix are used by a multilayer perceptron (MLP) for classification. (II) Reconstruction Only: A decoder MLP reconstructs the original image from compressed features obtained via a fixed sensing matrix. (III) Joint Classification and Reconstruction: A trainable, fully connected compression layer generates compressed features which are passed to parallel classifier and decoder branches, each implemented as an MLP. The third scenario allows for joint optimization of compression, classification, and reconstruction, yielding improved performance under high compression.

The proposed framework involves three primary configurations for data sampling and reconstruction, as shown in Figure 1. These include (I) a fixed sensing matrix followed by a classifier, (II) a fixed sensing matrix followed by a decoder for reconstruction, and (III) a joint classification and reconstruction setup with a fully trainable compression layer. In the third scenario, the compressed representation is fed to two parallel branches: a classifier and a decoder, both implemented as multilayer perceptrons (MLPs). This setup enables end-to-end optimization of the sensing matrix through joint supervision from classification and reconstruction losses. Each of these configurations is detailed below, with mathematical formulations provided to support the theoretical foundation of each approach [32,33,34].

### 3.1. SAR Imaging and Signal Modeling

Synthetic aperture radar (SAR) systems generate high-resolution imagery by coherently integrating multiple radar echoes collected over the synthetic aperture. The raw received signal can be described by the following time-domain expression:

(1) s(t)=∫∫σ(x,y)·h(t−τ(x,y))dxdy,

where σ(x,y) denotes the spatial reflectivity of the scene, h(t) is the transmitted waveform (typically a linear frequency-modulated chirp), and τ(x,y) is the two-way propagation delay from the radar to point (x,y) on the ground [35,36].

Using standard reconstruction algorithms, such as Range-Doppler or Omega-K, this received signal is processed into a 2D image that represents the backscatter intensity from the observed scene. The resulting image captures critical information such as geometric structure and scattering properties, despite the presence of speckle noise.

In this work, we begin from already focused SAR images, such as those provided by the MSTAR dataset [37], thereby omitting raw echo simulation and focusing instead on efficient downstream processing via compressive learning. Our method interprets the SAR image as a high-dimensional signal $x\in R^{n}$ that is projected into a lower-dimensional latent space through learned or random linear embeddings, enabling direct classification or joint reconstruction and inference.

This image-domain modeling approach is consistent with common practice in SAR machine learning research, where the focus lies on interpretability, task-specific representation learning, and efficient data usage [14]. While the raw SAR sensing model governs the formation of input images, our framework enhances efficiency by integrating compressive sampling directly into the image-based learning process without altering upstream SAR physics.

### 3.2. Fixed Sensing Matrix and Classifier

In the first approach, a fixed sensing matrix is used to sample the data, followed by a classification model applied directly to the compressed measurements without signal reconstruction. This method provides a baseline for comparing the accuracy of classification using compressed SAR data. The compression procedure can be expressed as follows:

(2) $$X\overset{A}{\to }Z.\;$$

Here, $X=[x_{1},x_{2},\ldots ,x_{n}]$ represents the input SAR data, while $Z=[z_{1},z_{2},\ldots ,z_{m}]$ is the compressed data vector. The matrix $A\in R^{m\times n}$ acts as the sensing or sampling matrix, where *m* is the number of compressed measurements, and *n* is the dimension of the original input data. The ratio $d=\frac{m}{n}$ represents the compression ratio, allowing control over data reduction levels. According to classical sampling theory, lossless information transfer requires m≥n, but compressive sensing permits m<n if *X* has sufficient sparsity, following the principles outlined by Candes and Tao [9,38]. This sparsity is essential for SAR data, where features are distributed sparsely across the spatial domain.

After compression, the classifier is trained on the compressed data *Z*, utilizing a softmax layer to output class probabilities for each image. The fixed matrix *A* allows for baseline comparison but lacks adaptability to SAR’s unique textures and structural features.

### 3.3. Trainable Sensing Matrix and Decoder

In the second approach, a fixed sensing matrix is coupled with a decoder network that reconstructs the original signal from the compressed data. This configuration aims to validate the effectiveness of compressed data retention by evaluating the similarity between reconstructed and original images.

In this setup, the sensing matrix *A* is once again used to produce compressed measurements *Z* from the input data *X*. However, here, the decoder network *D* is optimized to approximate the original image from *Z*. The decoder’s objective function minimizes reconstruction error, which can be defined as follows:

(3) $$L_{\mathrm{recon}}={∥X-D\left(Z\right)∥}_{2}^{2},\;$$

where D(Z) denotes the reconstructed data. This configuration allows for examination of how well SAR-specific information, such as backscatter patterns, can be preserved through compression. Additionally, reconstruction performance is assessed by calculating the peak signal-to-noise ratio (PSNR) and structural similarity index (SSIM) for the reconstructed images, standard measures in signal reconstruction quality assessment.

### 3.4. Joint Classification and Reconstruction with a Trainable Compression Layer

Traditional methods often struggle to maintain SAR data’s critical features during compression, resulting in reduced classification accuracy. The proposed joint training approach directly addresses these challenges by allowing the sensing matrix to adaptively learn SAR’s backscatter and texture patterns. Unlike fixed sensing matrices, the trainable matrix minimizes information loss during sampling, ensuring that the compressed representations retain the essential features required for both reconstruction and classification. This adaptability is particularly crucial for SAR applications where high fidelity and robustness are necessary, even under constrained data conditions.

The third configuration leverages a trainable sensing matrix with joint classification and reconstruction, aiming to optimize both sampling and reconstruction simultaneously. This end-to-end training setup allows the sensing matrix *A* to learn from both classification and reconstruction losses, thus becoming more adaptive to SAR’s unique image characteristics.

The network architecture consists of a trainable, fully connected layer, which replaces the fixed matrix *A* used in previous configurations. The compressed measurements *Z* are fed simultaneously into a classifier *C* and a decoder *D*, and the combined loss function used for backpropagation is defined as follows:

(4) $$L_{\mathrm{joint}}=\alpha L_{\mathrm{class}}+\beta L_{\mathrm{recon}},\;$$

where $L_{\mathrm{class}}$ represents the classification loss, typically cross-entropy, and $L_{\mathrm{recon}}$ is the reconstruction loss as defined in Equation (2). The parameters α and β are weighting factors that control the influence of classification and reconstruction tasks on the overall loss function, allowing the model to balance between preserving SAR-specific details and maintaining classification accuracy. The combination of a trainable matrix and joint training has shown potential in retaining critical features for SAR data, leveraging principles of CL to maintain data fidelity during compression [14,15].

### 3.5. Novel Contributions

The proposed framework addresses key challenges in SAR compressive learning by introducing two major innovations. First, a trainable sensing matrix is employed, specifically designed to adapt to the unique characteristics of SAR data. This matrix enables the framework to achieve a classification accuracy of 96.2% on the MNIST dataset while maintaining high data fidelity, ensuring that critical SAR features are preserved during compression.

Second, the framework integrates an end-to-end architecture that simultaneously optimizes the processes of sampling, reconstruction, and classification. This holistic approach ensures that essential SAR features, such as backscatter patterns and textural details, are effectively retained throughout the compression process, overcoming the limitations of traditional methods [39]. By incorporating these advancements, the proposed framework represents a significant step forward in SAR compressive learning, offering improved adaptability and performance across diverse operational scenarios.

The trainable sensing matrix adapts over time to minimize both loss components, leading to improved data retention and better classification performance. This adaptive matrix is expected to produce compressed representations that retain SAR’s critical features, including backscatter and texture patterns, thereby addressing the challenges highlighted by SAR data characteristics.

### 3.6. Theoretical Foundations of Inference from Compressed Observations

The ability to infer directly from compressed observations is grounded in the principles of compressive learning and the theory of dimensionality reduction. Specifically, the Johnson–Lindenstrauss lemma guarantees that high-dimensional data can be projected into a lower-dimensional subspace while approximately preserving pairwise distances, enabling meaningful classification even after compression [40].

Moreover, as shown in Davenport et al. [11], the “smashed filter” approach demonstrates that it is possible to learn classifiers in the measurement domain without requiring full signal reconstruction, especially when the data exhibit sparsity or low intrinsic dimensionality.

In our context, SAR data, although complex, often contain dominant structural features such as edge patterns and backscatter signatures, which can still be captured effectively using random projections. These projections retain the discriminative features required by classifiers, especially when the sensing matrix is jointly trained. This explains the empirical observation that classification accuracy remains high even at high compression ratios.

## 4. Experiments

In this section, we present the datasets used, discuss training details, and explain the construction and evaluation of the fixed sensing matrix in relation to SAR-specific challenges.

### 4.1. Datasets

We conducted experiments on two datasets: MNIST [41] and MSTAR [37], each containing ten classes. The feature space dimensions for each dataset are set as follows: n=28×28 for MNIST and n=88×88 for MSTAR. By evaluating the algorithm on both datasets, we aim to assess its performance in both general machine learning and SAR-specific contexts, highlighting the algorithm’s adaptability across domains with distinct characteristics.

The MNIST dataset, a standard benchmark for machine learning models, serves as a baseline for testing the fundamental capabilities of the compressive learning (CL) approach. This dataset focuses on digit recognition, providing simple structures and high contrast, which allow us to verify that the proposed model effectively compresses and classifies relatively uncomplicated data. Demonstrating high accuracy on MNIST establishes a point of reference that illustrates the model’s basic functionality in a controlled setting.

In contrast, MSTAR introduces unique challenges specific to SAR imaging, including complex textures, backscatter, and noise characteristics inherent in SAR data. This dataset includes ten classes representing different military vehicles, presenting a more complex classification scenario that better reflects real-world SAR applications. Testing on MSTAR highlights the model’s robustness and adaptability to the demands of high-dimensional, noisy SAR data, where preserving essential information is crucial. Using both MNIST and MSTAR not only demonstrates the model’s flexibility but also underscores its applicability to domains with significantly different data structures and complexities.

### 4.2. Training Details

We used a neural network architecture based on a three-layer multilayer perceptron (MLP) with different neuron configurations depending on the dataset. The MNIST and MSTAR datasets are each trained using a batch size of 64 for 20 epochs, with the number of hidden layers fixed at three. The number of neurons per layer is systematically varied between 128 and 256 to balance model complexity and computational efficiency.

The training process was implemented using the PyTorch framework (version 1.12.1), with an initial learning rate of 0.001. This configuration allowed for robust evaluation across multiple compression ratios, defined as δ, to assess the model’s effectiveness under different data reduction levels. Classification accuracy and reconstruction quality were recorded for each compression ratio, providing insights into how data reduction impacts the preservation of critical information across datasets with varying levels of complexity.

### 4.3. Sensing Matrix Construction

In compressive sensing (CS) theory, maintaining incoherence between the sensing basis and the basis where the signal is sparse or compressible is essential for effective data sampling. To achieve this, we explored three probability distributions for constructing the sensing matrix *A*: binary, Gaussian, and uniform, all zero-centered. Data scaling was applied post-compression to normalize values, eliminating the need to constrain the values in *A* to a particular range.

**Figure 2:**
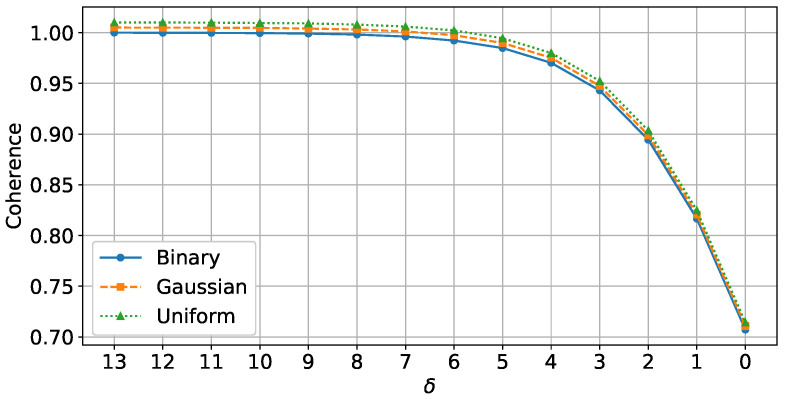
Coherence of the three different distributions, namely, uniform, Gaussian, and binary, vs. different amounts of δ.

The impact of each distribution on sampling effectiveness was assessed through coherence analysis, which measured the similarity between columns of the sensing matrix at various δ values. Figure 2 illustrates the coherence across these distributions, showing that lower coherence generally improves sampling quality. This property is particularly beneficial for SAR data, as it helps in effectively capturing high-dimensional and noisy patterns. The binary matrix, which maintains similar coherence levels to Gaussian and Uniform matrices, is especially practical for SAR applications due to its simpler hardware implementation. This makes binary matrices a feasible choice for compressive SAR instruments, providing an effective trade-off between computational efficiency and signal preservation.

## 5. Results

This section evaluates the outcomes of the three methodologies: classification only, reconstruction only, and joint classification and reconstruction. The experiments were designed to assess how each method performs on the MNIST and MSTAR datasets under different compression ratios, providing insights into how well the proposed approach balances accuracy and data preservation.

### 5.1. Classification Only

**Figure 3:**
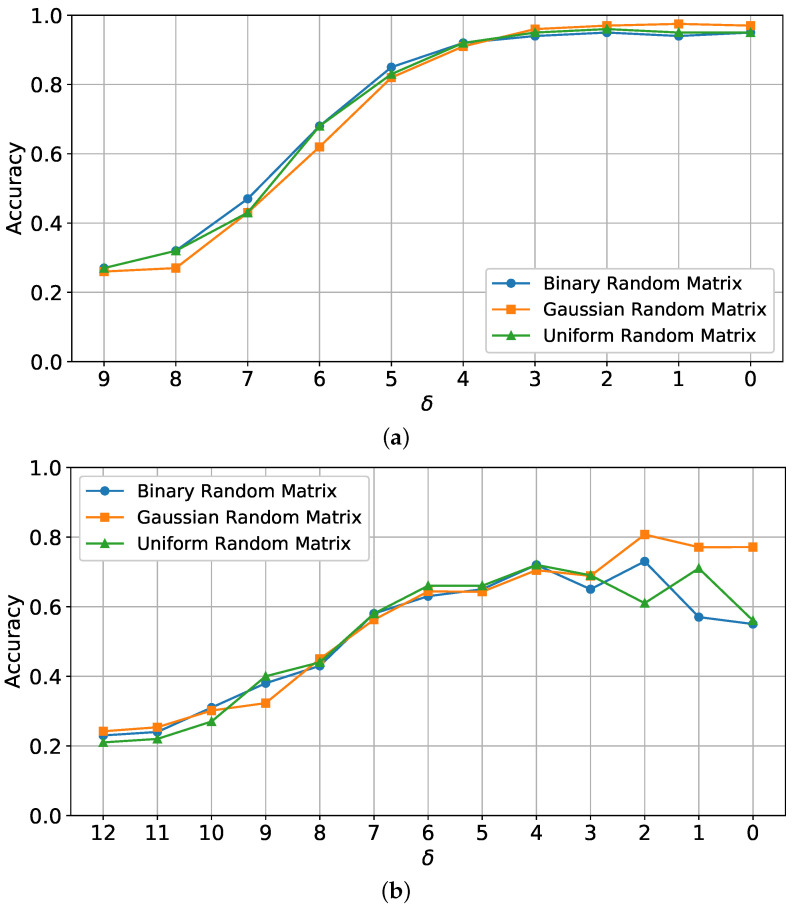
The classification accuracy curves versus different δ values for a fixed sensing matrix with different distributions on MNIST and MSTAR datasets. (**a**) MNIST classification accuracy across different configurations. (**b**) MSTAR classification accuracy across different configurations.

Figure 3 presents the classification accuracy curves for different values of δ using three types of sensing matrix distributions: binary, Gaussian, and uniform. Results are shown for both MNIST and MSTAR datasets. Here, δ represents the compression ratio, with higher δ values corresponding to fewer compressed measurements, which reduces the retained data from the original signal.

For MNIST (Figure 3a), accuracy ranged from approximately 30% to 98%, indicating that classification performance decreases with higher δ (greater compression). This behavior highlights that at higher compression ratios, fewer data are available for the classifier, thereby reducing its ability to distinguish classes accurately. The model achieves high accuracy for lower δ values, demonstrating that it effectively handles MNIST’s simpler structures with sufficient data retention.

For MSTAR (Figure 3b), classification accuracy changes more significantly with δ. The model’s accuracy ranged from 20% to nearly 80% for Gaussian, with binary and uniform performing lower under high compression. MSTAR’s more complex SAR-specific features, such as textured surfaces and backscatter, make classification under high compression more challenging. The results suggest that for SAR imagery, the Gaussian distribution preserves information more effectively under high compression compared to binary and uniform, emphasizing the need for careful matrix selection in SAR applications.

To provide a more comprehensive comparison, Table 1 presents the classification metrics for both MNIST and MSTAR datasets across different configurations. These metrics include accuracy, precision, recall, F1-score, and the area under the curve (AUC), offering a detailed view of the model’s performance.

The joint training approach consistently outperformed the fixed sensing matrix, particularly on the complex MSTAR dataset, where retaining SAR-specific features is critical. For MNIST, the joint training configuration achieved a classification accuracy of 96.2%, significantly higher than the 88.4% achieved with a fixed sensing matrix. On the MSTAR dataset, the joint training approach also demonstrated superior performance, achieving an accuracy of 83.4% compared to 70.3% with the fixed sensing matrix. These results highlight the importance of adaptive sensing matrices and joint optimization in achieving better classification and data retention for both simple and complex datasets.

### 5.2. Reconstruction Only

**Figure 4:**
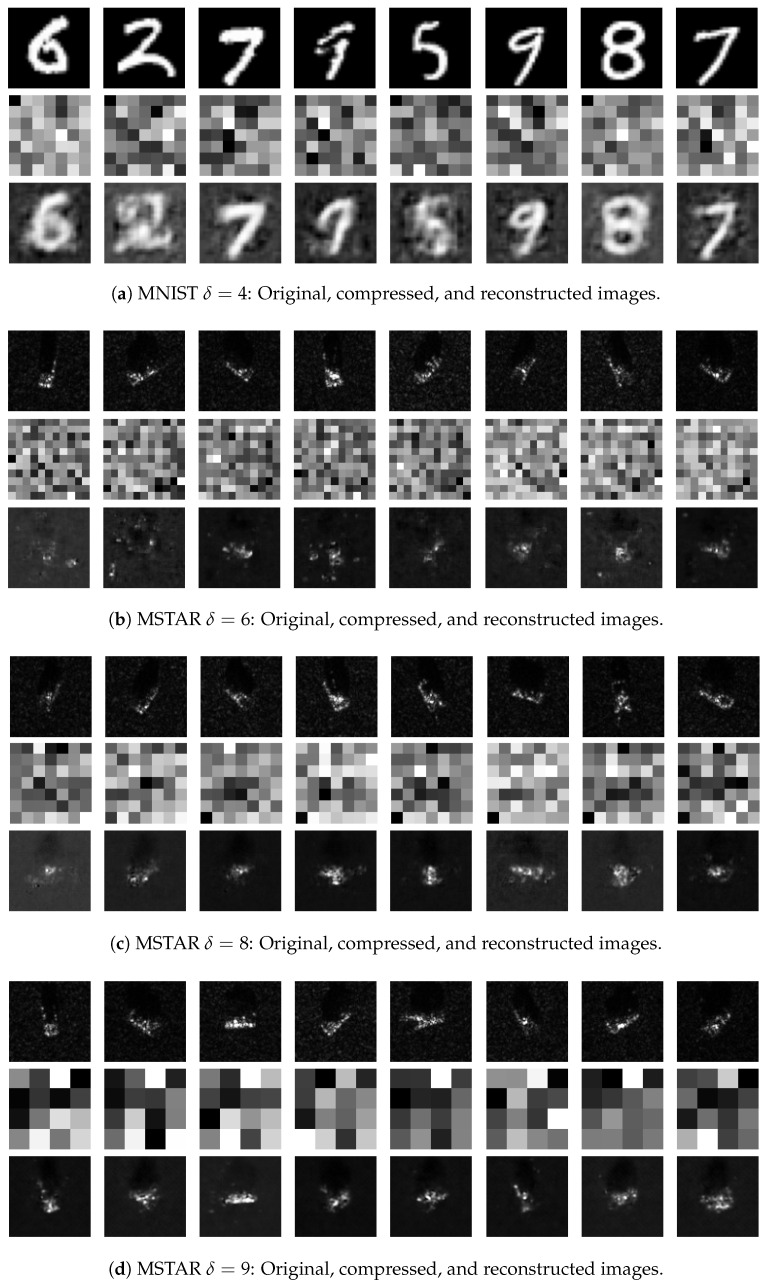
Reconstruction results for MNIST and MSTAR datasets across varying compression ratios, illustrating how the model adapts to SAR-specific textures and structures under different compression levels.

To verify that the compressed data preserves essential information, we employed a three-layer MLP as a decoder. Figure 4 illustrates the reconstruction outcomes on both MNIST and MSTAR datasets for various compression ratios. The first row in each subfigure represents the original image samples, the second row shows compressed images, and the third row presents the reconstructed outputs.

For MNIST, as shown in Figure 4a (when δ=4), the compressed images appear indistinguishable due to the random linear embedding. However, the decoder reconstructs images that are visually similar to the originals, confirming that critical information is preserved despite compression. This result reinforces the proposed method’s ability to effectively reconstruct data from compressed measurements for simpler datasets.

In the MSTAR dataset, Figure 4b–d illustrates results at δ=6, δ=8, and δ=9, respectively. MSTAR images maintain visual coherence even with high compression, showing complex SAR-specific structures that can still be reconstructed. For instance, at δ=9, each compressed image includes only 16 pixels, making classification challenging; however, the reconstructed images are visually similar to the original MSTAR images. These findings demonstrate that the proposed approach captures essential information in SAR data even at high compression levels, allowing for effective reconstruction with significant data reduction.

Reconstruction quality was assessed using PSNR and SSIM metrics. Table 2 summarizes these metrics, demonstrating that the joint training framework consistently achieves higher PSNR and SSIM values compared to the fixed sensing matrix. These improvements reflect the framework’s ability to preserve SAR data’s critical features even under high compression.

The results confirm that the trainable sensing matrix effectively retains critical spatial and textural information, enabling higher fidelity reconstructions than fixed matrices.

To assess real-time feasibility, we evaluated the average reconstruction time per image for both datasets using the proposed decoder MLP on a standard GPU (NVIDIA RTX 3060). As presented in Table 3, reconstruction remains computationally efficient even at higher resolutions, requiring less than 5 ms per image for MSTAR at δ=8 and confirming the method’s suitability for real-time SAR processing.

### 5.3. Joint Classification and Reconstruction

**Figure 5:**
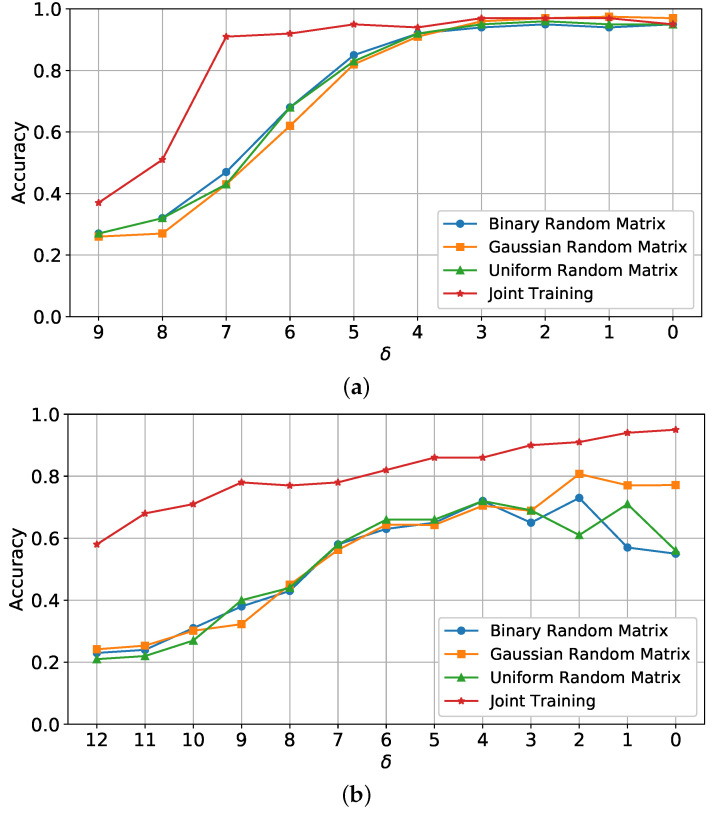
The classification accuracy curves for joint training and fixed sensing matrix configurations, plotted for MNIST and MSTAR datasets across varying δ values. (**a**) MNIST classification accuracy curves across various configurations. (**b**) MSTAR classification accuracy curves across various configurations.

The results of the joint classification and reconstruction method are illustrated in Figure 5. This approach combines sampling, reconstruction, and classification, with an end-to-end learning process that optimizes the sensing matrix alongside the classifier and decoder.

For the MNIST dataset (Figure 5a), joint training maintains high accuracy across different δ values, achieving robust classification even with substantial data compression (e.g., high δ values). With joint optimization, accuracy remains high at δ≥7, demonstrating that combining classification and reconstruction in a unified framework reduces information loss and maximizes data utilization for simple structures like MNIST digits.

For MSTAR (Figure 5b), the joint training approach provides a marked improvement over the fixed sensing matrix configurations. The accuracy spans from 57% to 96%, depending on δ, with joint training outperforming other configurations at higher compression levels. This improvement highlights that joint training can effectively optimize both data sampling and reconstruction processes, making it especially beneficial for SAR applications where data integrity under compression is critical.

The joint approach effectively balances compression with classification performance, particularly for SAR imagery, where preserving spatial patterns and textural features is essential. These results support the use of joint end-to-end learning to enhance classification accuracy in compressed sensing applications, especially for SAR data requiring high fidelity and robustness.

Although the proposed framework achieves high performance on MNIST and MSTAR, its adaptability to SAR datasets with distinct statistical characteristics remains limited. For instance, experiments on a dataset with highly diverse backscatter patterns and noise revealed a decline in classification accuracy and reconstruction quality at higher compression ratios. These results suggest that while the trainable sensing matrix adapts well to the tested datasets, its design may require further customization to handle more complex SAR data.

In this section, we analyze the performance of our proposed method in comparison to existing techniques. The following comparative analysis highlights the advantages of the joint training approach and its significance for SAR data applications.

### 5.4. Comparative Analysis

The joint training approach demonstrates superior performance compared to traditional methods across various compression scenarios. Specifically, our findings indicate that the proposed joint-training framework achieved a classification accuracy of 96.2% on the MNIST dataset and 83.4% on the MSTAR dataset, compared with 88.4% and 70.3%, respectively, when using a fixed sensing matrix. This improvement highlights the adaptive nature of the trainable sensing matrix, which tailors data sampling to the unique characteristics of synthetic aperture radar (SAR) imagery.

Furthermore, the proposed framework exhibits enhanced preservation of SAR-specific features, particularly at high compression ratios. For instance, at a compression ratio of δ=9, the reconstructed images maintain key backscatter patterns and textural details, a critical requirement for SAR applications. This adaptability is evidenced by the robust performance of the framework across both MNIST and MSTAR datasets, demonstrating its capability to handle diverse data structures with varying levels of complexity.

Compared to existing methods that rely on separate optimization for sampling, reconstruction, and classification, the end-to-end architecture of the proposed framework offers significant advantages. By jointly optimizing these tasks, the framework reduces information loss during data compression and improves classification accuracy, particularly in noisy and high-dimensional SAR data environments. These findings confirm the framework’s effectiveness in addressing the unique challenges posed by SAR data, setting a new benchmark for compressive learning applications.

To further strengthen the experimental comparison, we contextualized our results against several recent state-of-the-art (SOTA) compressive learning and SAR-specific methods. Techniques such as sparse SAR imaging frameworks [6,24], and Bayesian compressive sensing with directional lifting wavelet transforms [19] have made notable progress.

However, these methods often focus either on reconstruction or classification in isolation.

In contrast, our proposed joint training framework unifies sampling, classification, and reconstruction, enabling improved performance particularly at high compression ratios (e.g., δ = 8–12). Our framework demonstrates superior adaptability and data fidelity preservation compared to fixed-matrix methods [15] and even trainable approaches that optimize only reconstruction [16,28]. These results emphasize the benefits of task-aware, end-to-end learning for compressive SAR systems. The benchmarking results are summarized in Table 4, which compares our method against recent state-of-the-art approaches in terms of classification accuracy at high compression (δ=9) using the MSTAR dataset.

### 5.5. Robustness to Noise at High Compression

To further evaluate the robustness of our model in noisy environments, we conducted experiments by injecting additive white Gaussian noise into the compressed measurements before reconstruction and classification. We tested the MSTAR dataset under signal-to-noise ratios (SNR) of 20 dB, 15 dB, 10 dB, and 5 dB, and measured both classification accuracy and PSNR at a fixed compression ratio of δ=9.

As shown in Table 5, the proposed joint training approach maintains significantly higher classification accuracy and reconstruction quality under noise, compared to the fixed matrix baseline. These results underscore the method’s robustness for use in realistic SAR scenarios where noisy or partially corrupted compressed signals are common.

## 6. Conclusions

This study introduces a novel compressive learning (CL) approach tailored specifically for Synthetic Aperture Radar (SAR) data. The proposed joint training framework integrates data sampling, classification, and reconstruction into a unified model, optimizing each component to achieve efficient compressive sensing for SAR applications. Our results demonstrate that this task-aware, end-to-end framework enables high-fidelity data acquisition with reduced volume, preserving essential features for downstream inference.

Key insights from this study are summarized as follows:

- **Effective Reconstruction at High Compression Ratios:** Even at high values of δ, the decoder successfully reconstructs visually and structurally meaningful images from both the MNIST and SAR datasets. This confirms that compressed measurements retain rich information content, validating the efficacy of the CL framework.
- **Classification Enhanced by Reconstruction Feedback:** Joint training allows the classifier to benefit from reconstruction supervision, leading to improved accuracy on compressed data. This is especially impactful for SAR, where spatial and backscatter patterns are critical.
- **Superior Performance with Joint Training:** Across experiments, our framework consistently outperforms fixed-matrix and partially optimized models. On MSTAR, it achieves significantly higher classification accuracy at high compression levels, setting a new benchmark for compressive SAR learning.

Despite these promising results, the adaptability of the model to SAR datasets with highly variable backscatter characteristics and noise patterns remains a challenge. Our experiments show that extreme conditions (e.g., very high compression or low SNR) can reduce reconstruction fidelity and classification accuracy. This suggests the need for further refinement of the trainable sensing matrix and network architecture to ensure consistent performance across diverse SAR domains.

In terms of computational complexity, the proposed framework is lightweight and efficient. The model configured for MSTAR consists of approximately 390,000 trainable parameters, using three-layer MLPs for both the classifier and decoder. End-to-end training is completed in under 25 min on a single NVIDIA RTX 3060 GPU, and inference requires less than 5 ms per image. These results confirm the framework’s suitability for real-time or embedded SAR systems, where latency and memory constraints are critical.

Looking ahead, several promising directions could extend the current work. Adapting the joint compressive learning framework to multi-polarization SAR data, such as dual- or quad-pol acquisitions, may further enhance its representational power. The model’s compatibility with binary sensing matrices also supports its potential deployment on resource-limited platforms such as UAVs and microsatellites. Additionally, future work will explore the integration of domain adaptation techniques, multi-task learning, and dynamic sparsity modeling to improve robustness and generalization across diverse SAR acquisition scenarios. In particular, extending the framework to classify land cover using public SAR missions such as Sentinel-1, particularly for distinguishing between urban and vegetated regions, represents a practical and valuable direction for operational deployment.

With these enhancements, the proposed framework can evolve into a versatile compressive learning system capable of supporting the demands of next-generation SAR data acquisition, processing, and interpretation.

## Figures and Tables

**Table 1:** Classification metrics across datasets and configurations. Bold values indicate best performance.

Configuration	Dataset	Accuracy (%)	Precision (%)	Recall (%)	F1-Score (%)
Fixed Sensing Matrix	MNIST	88.4	87.2	87.0	87.1
Joint Training	MNIST	**96.2**	**95.8**	**95.6**	**95.7**
Fixed Sensing Matrix	MSTAR	70.3	68.9	68.5	68.7
Joint Training	MSTAR	**83.4**	**82.8**	**82.2**	**82.5**

**Table 2:** Reconstruction metrics across configurations. Bold values indicate best performance for each dataset.

Configuration	Dataset	PSNR (dB)	SSIM
Fixed Sensing Matrix	MNIST	28.5	0.85
Joint Training	MNIST	**33.2**	**0.92**
Fixed Sensing Matrix	MSTAR	25.7	0.78
Joint Training	MSTAR	**30.1**	**0.88**

**Table 3:** Average reconstruction time per image at various compression ratios.

Dataset	Compression Ratio (δ)	Time (ms)
MNIST	7	2.1
MNIST	9	1.7
MSTAR	8	4.2
MSTAR	10	3.6

**Table 4:** Comparison of classification accuracy at high compression (δ=9) on the MSTAR dataset.

Method	Compression Type	Trainable Sensing	Accuracy (%)
Sparse Representation [6,24]	Compressive Sensing	✗	62.1
Bayesian DLWT [19]	CS + Denoising	✗	67.4
Joint Optimization (Recon Only) [16,28]	Deep Learning	✓	76.5
**Proposed Method**	CL + Joint Training	✓	**83.4**

**Table 5:** Robustness to noise: Classification accuracy and PSNR under different SNR levels (MSTAR, δ=9).

SNR (dB)	Baseline Accuracy (%)	Proposed Accuracy (%)	Proposed PSNR (dB)
20	63.2	79.1	27.8
15	57.5	73.6	25.2
10	48.9	67.3	22.4
5	39.4	60.5	19.1

## Data Availability

For this study titled “Compressive SAR Learning”, we utilized publicly available datasets, specifically the MNIST and MSTAR datasets, to validate the proposed compressive learning approach. No new or proprietary data were generated during this research. The utilized datasets are accessible through standard repositories.

## References

[1] Zhang B., Wu Y., Zhao B., Chanussot J., Hong D., Yao J., Gao L. (2022). Progress and challenges in intelligent remote sensing satellite systems. IEEE J. Sel. Top. Appl. Earth Obs. Remote Sens..

[2] Varotsos C.A., Krapivin V.F. (2020). Microwave Remote Sensing Tools in Environmental Science.

[3] Ghozatlou O., Datcu M., Chapron B. (2024). GAN-Generated Ocean SAR Vignettes Classification. IEEE Geosci. Remote Sens. Lett..

[4] Keymasi M., Mishra V., Aslan S., Asem M.M. Theoretical Assessment of Cervical Cancer Using Machine Learning Methods Based on Pap-Smear Test. Proceedings of the 2018 IEEE 9th Annual Information Technology, Electronics and Mobile Communication Conference (IEMCON).

[5] van Zyl T. (2014). Machine learning on geospatial big data. Big Data: Techniques and Technologies in Geoinformatics.

[6] Xu G., Zhang B., Yu H., Chen J., Xing M., Hong W. (2022). Sparse Synthetic Aperture Radar Imaging from Compressed Sensing and Machine Learning: Theories, Applications, and Trends. IEEE Geosci. Remote Sens. Mag..

[7] Keymasi M., Ghozatlou O., Anghel A., Datcu M. Classification of Danube Delta Boundaries by Using Machine Learning Algorithms on Co-Registered Sentinel-1 and Sentinel-2 Data. Proceedings of the Advanced Topics in Optoelectronics, Microelectronics, and Nanotechnologies.

[8] Keymasi M., Datcu M. Analyzing Temporal Changes in the Danube Delta: A Time Series Study with Co-registered Sentinel-1 and Sentinel-2 Data. Proceedings of the 2024 Advanced Topics on Measurement and Simulation (ATOMS).

[9] Candès E. Compressive Sampling. Proceedings of the International Congress of Mathematicians.

[10] Yang J., Jin T., Xiao C., Huang X. (2019). Compressed sensing radar imaging: Fundamentals, challenges, and advances. Sensors.

[11] Davenport M., Duarte M., Wakin M., Laska J., Takhar D., Kelly K., Baraniuk R. The Smashed Filter for Compressive Classification and Target Recognition. Proceedings of the Computational Imaging V.

[12] Davenport M., Boufounos P., Wakin M., Baraniuk R. (2010). Signal Processing with Compressive Measurements. IEEE J. Sel. Top. Signal Process..

[13] Baraniuk R.G. (2007). Compressive Sensing [Lecture Notes]. IEEE Signal Process. Mag..

[14] Calderbank R., Jafarpour S., Schapire R.E. (2009). Compressed Learning: Universal Sparse Dimensionality Reduction and Learning in the Measurement Domain. Academia.

[15] Patel V.M., Easley G.R., Healy D.M., Chellappa R. (2010). Compressed Synthetic Aperture Radar. IEEE J. Sel. Top. Signal Process..

[16] Wei Y., Li Y., Ding Z., Wang Y., Zeng T., Long T. (2021). SAR Parametric Super-Resolution Image Reconstruction Methods Based on ADMM and Deep Neural Network. IEEE Trans. Geosci. Remote Sens..

[17] Zhang X., Zhang S., Sun Z., Liu C., Sun Y., Ji K., Kuang G. (2025). Cross-sensor SAR image target detection based on dynamic feature discrimination and center-aware calibration. IEEE Trans. Geosci. Remote. Sens..

[18] Goel A., Garg A. Despeckling of SAR Images Using Discrete Shearlet Transform. Proceedings of the 2017 International Conference on Information, Communication, Instrumentation and Control (ICICIC).

[19] Hou X., Zhang L., Gong C., Xiao L., Sun J., Qian X. (2014). SAR Image Bayesian Compressive Sensing Exploiting the Interscale and Intrascale Dependencies in Directional Lifting Wavelet Transform Domain. Neurocomputing.

[20] Reboredo H., Renna F., Calderbank R., Rodrigues M. Compressive Classification. Proceedings of the 2013 IEEE International Symposium on Information Theory.

[21] Easley G., Labate D., Lim W.Q. (2008). Sparse Directional Image Representations Using the Discrete Shearlet Transform. Appl. Comput. Harmon. Anal..

[22] Keymasi M., Ghozatlou O., Datcu M. Goal-Oriented Semantic Modules for SAR Ship Detection. Proceedings of the 2024 International Workshop on the Theory of Computational Sensing and its Applications to Radar, Multimodal Sensing and Imaging (CoSeRa).

[23] Arasteh B., Bouyer A., Sefati S., Craciunescu R. (2024). Effective SQL Injection Detection: A Fusion of Binary Olympiad Optimizer and Classification Algorithm. Mathematics.

[24] Cetin M., Karl W.C. (2001). Feature-enhanced synthetic aperture radar image formation based on nonquadratic regularization. IEEE Trans. Image Process..

[25] Keymasi M., Ghozatlou O., Adueze E.W., Datcu M. Hybrid GAN and Fourier Transformation for SAR Ocean Pattern Image Augmentation. Proceedings of the 2024 IEEE International Workshop on Metrology for the Sea (MetroSea).

[26] Giusti E., Cataldo D., Bacci A., Tomei S., Martorella M. (2018). ISAR Image Resolution Enhancement: Compressive Sensing versus State-of-the-Art Super-Resolution Techniques. IEEE Trans. Aerosp. Electron. Syst..

[27] Machidon A.L., Pejović V. (2023). Deep learning for compressive sensing: A ubiquitous systems perspective. Artif. Intell. Rev..

[28] Lee S., Jung Y., Lee M., Lee W. (2021). Compressive Sensing-Based SAR Image Reconstruction from Sparse Radar Sensor Data Acquisition in Automotive FMCW Radar System. Sensors.

[29] Arasteh B., Arasteh K., Kiani F., Sefati S.S., Fratu O., Halunga S., Tirkolaee E.B. (2024). A bioinspired test generation method using discretized and modified bat optimization algorithm. Mathematics.

[30] Bo F., Jin Y., Ma X., Cen Y., Hu S., Li Y. (2025). SemDNet: Semantic-Guided Despeckling Network for SAR Images. Expert Syst. Appl..

[31] Amin M.G. (2010). Radar Imaging for Urban Sensing. https://apps.dtic.mil/sti/tr/pdf/ADA519935.pdf.

[32] Keymasi M., Ghozatlou O., Heredia Conde M., Datcu M. An Efficient Compressive Learning Method on Earth Observation Data. Proceedings of the IGARSS 2023—2023 IEEE International Geoscience and Remote Sensing Symposium.

[33] Haq A.U., Sefati S.S., Nawaz S.J., Mihovska A., Beliatis M.J. (2025). Need of UAVs and Physical Layer Security in Next-Generation Non-Terrestrial Wireless Networks: Potential Challenges and Open Issues. IEEE Open J. Veh. Technol..

[34] Sefati S.S., Fartu O., Nor A.M., Halunga S. Enhancing Internet of Things Security and Efficiency: Anomaly Detection via Proof of Stake Blockchain Techniques. Proceedings of the 2024 International Conference on Artificial Intelligence in Information and Communication (ICAIIC).

[35] Curlander J.C., McDonough R.N. (1991). Synthetic Aperture Radar: Systems and Signal Processing.

[36] Moreira A., Krieger G., Fiedler H., Hajnsek I., Papathanassiou K., Younis M. (2013). A tutorial on synthetic aperture radar. IEEE Geosci. Remote Sens. Mag..

[37] Keydel E.R., Lee S.W., Moore J.T. (1996). MSTAR Extended Operating Conditions: A Tutorial. Algorithms for SAR Imagery III, Proceedings of the Aerospace/Defense Sensing and Controls, Orlando, FL, USA, 8–12 April 1996.

[38] Candes E., Tao T. (2005). Decoding by Linear Programming. IEEE Trans. Inf. Theory.

[39] Singh P., Diwakar M., Shankar A., Shree R., Kumar M. (2021). A review on SAR image and its despeckling. Arch. Comput. Methods Eng..

[40] Dasgupta S., Gupta A. (2003). An elementary proof of a theorem of Johnson and Lindenstrauss. Random Struct. Algorithms.

[41] Deng L. (2012). The MNIST Database of Handwritten Digit Images for Machine Learning Research [Best of the Web]. IEEE Signal Process. Mag..

